# Complication rates of CT-guided transthoracic lung biopsy: meta-analysis

**DOI:** 10.1007/s00330-016-4357-8

**Published:** 2016-04-23

**Authors:** W. J. Heerink, G. H. de Bock, G. J. de Jonge, H. J. M. Groen, R. Vliegenthart, M. Oudkerk

**Affiliations:** 1 Center for Medical Imaging-North East Netherlands, University of Groningen, University Medical Center Groningen, Groningen, Netherlands; 2 Department of Radiology, University of Groningen, University Medical Center Groningen, Groningen, Netherlands; 3 Department of Epidemiology, University of Groningen, University Medical Center Groningen, Groningen, Netherlands; 4 Department of Pulmonary Medicine, University of Groningen, University Medical Center Groningen, Groningen, Netherlands

**Keywords:** Lung neoplasms, Meta-analysis, Biopsy, Pneumothorax, Computed tomography, X-Ray

## Abstract

**Objectives:**

To meta-analyze complication rate in computed tomography (CT)-guided transthoracic lung biopsy and associated risk factors.

**Methods:**

Four databases were searched from 1/2000 to 8/2015 for studies reporting complications in CT-guided lung biopsy. Overall and major complication rates were pooled and compared between core biopsy and fine needle aspiration (FNA) using the random-effects model. Risk factors for complications in core biopsy and FNA were identified in meta-regression analysis.

**Results:**

For core biopsy, 32 articles (8,133 procedures) were included and for FNA, 17 (4,620 procedures). Pooled overall complication rates for core biopsy and FNA were 38.8 % (95 % CI: 34.3–43.5 %) and 24.0 % (95 % CI: 18.2–30.8 %), respectively. Major complication rates were 5.7 % (95 % CI: 4.4–7.4 %) and 4.4 % (95 % CI: 2.7–7.0 %), respectively. Overall complication rate was higher for core biopsy compared to FNA (*p* < 0.001). For FNA, larger needle diameter was a risk factor for overall complications, and increased traversed lung parenchyma and smaller lesion size were risk factors for major complications. For core biopsy, no significant risk factors were identified.

**Conclusions:**

In CT-guided lung biopsy, minor complications were common and occurred more often in core biopsy than FNA. Major complication rate was low. For FNA, smaller nodule diameter, larger needle diameter and increased traversed lung parenchyma were risk factors for complications.

***Key Points*:**

• *Minor complications are common in CT-guided lung biopsy*

• *Major complication rate is low in CT-guided lung biopsy*

• *CT-guided lung biopsy complications occur more often in core biopsy than FNA*

• *Major complication rate is similar in core biopsy and FNA*

• *Risk factors for FNA are larger needle diameter, smaller lesion size*

**Electronic supplementary material:**

The online version of this article (doi:10.1007/s00330-016-4357-8) contains supplementary material, which is available to authorized users.

## Introduction

In the US, lung cancer screening by low-dose computed tomography (CT) is recommended for people at high risk [1], and the European Society of Radiology and the European Respiratory Society have recently recommended lung cancer screening within clinical trial setting or in routine clinical practice at certified medical centers [2]. This development will cause an increase in CT-detected lung nodules. Nodules >10 mm and most likely even smaller nodules with high growth rate will be eligible for medical work-up, including CT-guided lung biopsy [2]. CT-guided transthoracic lung biopsy is a minimally invasive diagnostic procedure for tissue diagnosis of peripheral lung nodules. This can alternatively be achieved by surgery, but CT-guided transthoracic lung biopsy is less invasive and associated with lower costs.

CT-guided transthoracic lung biopsy is a widely accepted procedure [3, 4], although the reported complication rate varies greatly. Where some papers report a higher complication rate for core needle biopsy compared to fine needle aspiration (FNA) [5, 6], other studies [7, 8] do not. Yao et al. [9] concluded in a systematic review comparing FNA with core biopsy that no significant difference in complication rate between these techniques exists. They also concluded that core biopsy is generally reported to have a somewhat higher diagnostic performance compared to FNA, especially in identifying histological subtypes; the evidence is insufficient to support a difference.

Complication rate and diagnostic performance are the two main factors in choosing a diagnostic procedure. To determine the role of CT-guided transthoracic lung biopsy in the work-up of screen-detected lung nodules, it is imperative to know how safe the procedure is. We conducted this meta-analysis to 1) determine the complication rate, and 2) identify risk factors for complications of CT-guided core biopsy and FNA.

## Materials and methods

This study was conducted according to the PRISMA guidelines for systematic reviews and meta-analyses [10].

### Search strategy and study selection

A literature research was performed from January 2000 to August 21, 2015, on PubMed, Embase, Web of Science, and the Cochrane Library using variations of the combination of the following search terms: (biopsy OR FNA) AND (transthoracic OR CT-guided) AND (lung cancer) AND CT. Please see e-Table 1 for the set of search terms per database. After screening title and abstract, two reviewers (W.H., G.J.) evaluated the full text of the remaining articles, with disagreements resolved by consensus.

Inclusion criteria were: (a) reporting of complications of at least 50 procedures; (b) differentiation in complications between core biopsy and FNA if both techniques were used; (c) the study was not a subset of patients from other included studies; (d) adequate complication monitoring. Complication monitoring was considered adequate if directly following the procedure a CT scan was acquired, plus a CT scan or chest radiograph 2 to 4 h after the procedure. Studies were excluded during screening if they clearly addressed a different topic, were case reports, conference abstracts, reviews or editorials, or if they were not published in English.

A standardized extraction form was used to collect the characteristics of the study regarding patients, nodules, procedures and complications, and how complication monitoring was performed. Two authors (W.H., G.J.) independently extracted these data, with disagreement resolved by consensus. The database was split according to the biopsy method, and all analyses were performed separately for core biopsy and FNA.

The methodological quality of the studies was assessed using the Newcastle-Ottawa Scale for nonrandomized studies, with results in a score of 0 to 9 [11]. Two authors (W.H., G.J.) independently scored the studies, with disagreement resolved by consensus.

Complications were classified as minor or major according to the Society of Interventional Radiology (SIR) Guidelines [12]. Minor complications consisted of pneumothorax without need for intervention, ground glass opacity around the target diagnosed as pulmonary hemorrhage, and transient hemoptysis. Major complications consisted of pneumothorax requiring intervention, hemothorax, air embolism, needle tract seeding, and death. Intervention was defined as treatment consequences (manual aspiration, chest tube placement, or pain control) or hospital admission. For each study the number of (major) complications was determined as the sum of all reported (major) complications. If the complications for different subgroups were reported, the number of (major) complications was determined per subgroup.

Study-specific risk factors for overall complications and for major complications that were examined are listed in Table 1.

**Table 1:** Study-specific characteristics examined as risk factors for (major) complications

Potential risk factor	Explanation
Mean nodule size	Greatest axial cross section of lesion (mm)
Mean nodule depth	Distance skin-lesion (mm)
	Distance pleura-lesion (mm)
Mean number of biopsies	Number of biopsy samples acquired per procedure
Use of coaxial needle	–
Biopsy needle diameter	When using coaxial needle, the coaxial needle diameter was used, as this is the outer diameter (mm)
Use of CT-fluoroscopy	–
Use of biopsy site down technique	Post-procedural repositioning of patients with the biopsy site facing downwards in an effort to reduce complication rate
Presence on-site cytopathology	–
Mean procedural time	–
Number of operators	–
Study size	–
Malignancy rate	Ratio of procedures in which the lesion was diagnoses as malignant by CT-guided biopsy of FNA
Operator experience	If an operator experience range was mentioned, the minimal mean operator experience was determined (i.e. four operators with 5–10 years experience resulted in a mean of 6.25 years)
Institute frequency	If all procedures were performed consecutively or with a negligible number of excluded cases (<10 %), and the inclusion start and end dates were reported, the frequency at which the procedure was performed at the institute was determined. A distinction was made between high volume centres, with one or more procedures per week, and low volume centres, less than one procedure per week.

### Data analysis

Heterogeneity in the overall complication rate between studies was tested and quantified using the *I* ^*2*^ index [13]. *I* ^*2*^ values of 0, 25, 50, and 75 % were defined as no, low, moderate, and high heterogeneity, respectively. To identify sources of heterogeneity, the effect of potential risk factors on between-study heterogeneity was investigated. The R^2^ equivalent was determined to express the true variance explained by the model, as a proportion of the total true variance. The risk of publication bias was visually assessed with funnel plots of major complication rate against the sample size of the individual studies.

Pooled complication rates for core biopsy and FNA were calculated using the random-effects model, weighted by the inverse variance of the studies, under the assumption of heterogeneity. Across subgroups, a common among-study variance component was assumed, and subgroups were combined using a fixed effect model. Forest plots were made for major complications and for all types of complications separately. Differences in complication rate between core biopsy and FNA procedures were assessed using regression analysis. All recorded study, patient, nodule, and procedural characteristics as listed in Table 1 were analyzed as potential risk factors for overall complications and for major complications in regression analysis. Correlations with significance level of *p* > 0.10 are presented with odds ratio (OR) and 95 % confidence interval (95 % CI). Statistical significance was set at *p* < 0.05. All statistical analyses were conducted with Comprehensive Meta-Analysis (CMA, version 3.2.070).

## Results

**Fig. 1:**
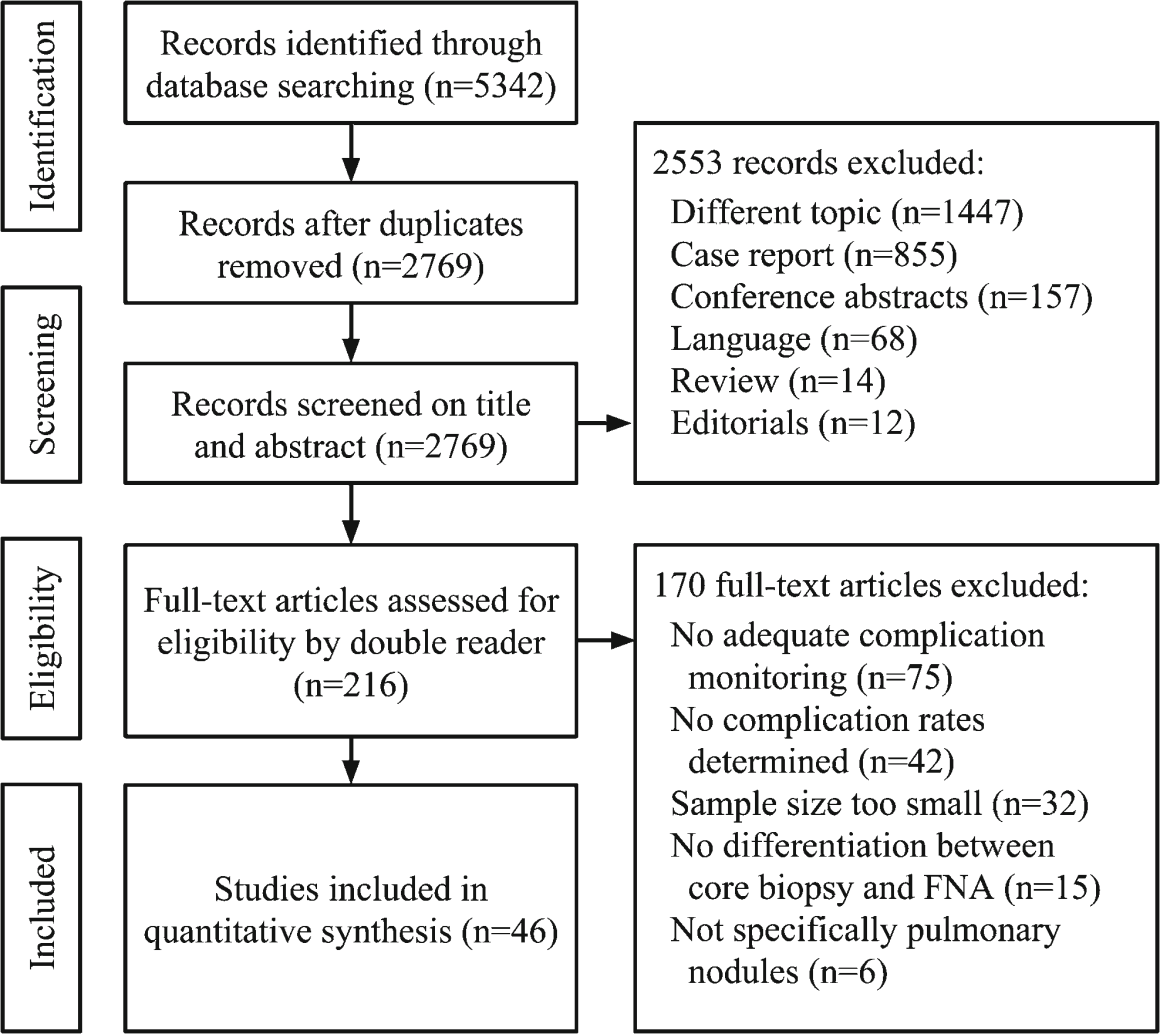
PRISMA flow chart of article selection process

Figure 1 shows the PRISMA flow diagram. Thirty-two included studies reported complications of core biopsy and 17 of FNA. Three studies reported complications of both biopsy techniques. Tables 2 [7, 8, 14–39] and 3 [7, 8, 40–53] show the study characteristics, complication rates, NOS scores, and forest plots for major complications of the studies for core biopsy and FNA, respectively. Forest plots for all types of complications separately can be found in e-Tables 2–9. The median NOS scores of core biopsy and FNA studies were 8 and 7 out of 9, respectively (*p* = 0.917). The case control studies (*n* = 27) scored least points for *representativeness of cases* (19/27) and for *comparability of cases and controls* (20/54). The cohort studies (*n* = 18) scored least points for *comparability of cohorts* (26/36). Tables 3 and 4 summarizes the study, patient, nodule, and procedural characteristics of the included studies.

**Table 2:**
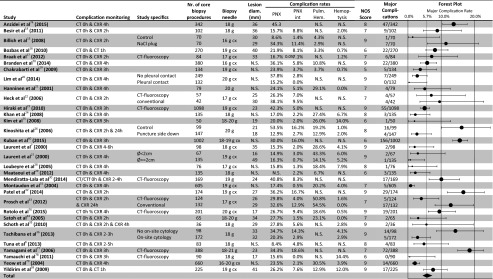
Characteristics, complication rates, and forest plot for major complications from included studies for core biopsy

*PNX*, pneumothorax; *PNX int*, pneumothorax requiring intervention; *NOS*, Newcastle-Ottawa Scale; *N.S.*, not specified; *g*, gauge; *cx*, coaxial needle; In the forest plot the major complication rate with 95 % CI is plotted, the size of the circles represents the weight of each individual (sub) study as assigned by the random effects model

**Table 3:**
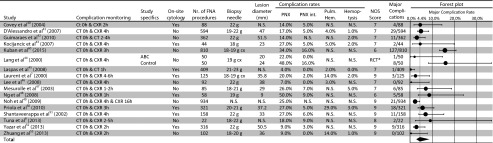
Characteristics, complication rates, and forest plot for major complications from included studies for FNA

*PNX*, pneumothorax; *PNX int*, pneumothorax requiring intervention; *NOS*, Newcastle-Ottawa Scale; *N.S.*, not specified; *g*, gauge; *cx*, coaxial needle; *ABC*, autologous blood clot; none of the included studies for FNA procedures used CT-fluoroscopy; *Randomized Controlled Trial: no NOS score could be determined. In the forest plot the major complication rate with 95 % CI is plotted, the size of the circles represents the weight of each individual (sub) study as assigned by the random effects model

**Table 4:** Study, procedural, patient, and nodule characteristics of included studies

	Core biopsy (*n* = 32)	FNA (*n* = 17)
Quality	Mean (SD) 7.6 (1.2)	Mean (SD) 7.7 (1.1)
	Median (range) 8 (5–9)	Median (range) 7 (6–9)
Procedures (*n*)	8,133	4,620
Needle gauge	18.2 (1.2)	21.3 (1.7)
Use of coaxial needle	25/29	4/16
Use of biopsy device	26/29	N.A.
Use of CT-fluoroscopy	10/29	0/16
Gender		
Male (n)	4,303	1,792
Female (n)	2,192	649
Patient age (years)	64.4 (2.9)	62.3 (3.8)
Pleural passes (*n*)	1.1 (0.4)	1.5
Nodule diameter (mm)	27.9 (7.8)	41.4 (10.0)
Distance skin-lesion (mm)	53.2 (13.3)	48.0 (6.0)
Traversed lung (mm)	16.6 (6.6)	14.1 (7.3)
Procedure time (min)	32 (5)	N.S.
Operator experience (years)	10.1 (4.0)	8.0 (2.5)

Data are presented as number of studies or means with standard deviation. Means are weighted by number of procedures. *N.A.* Not available

**Fig. 2:**
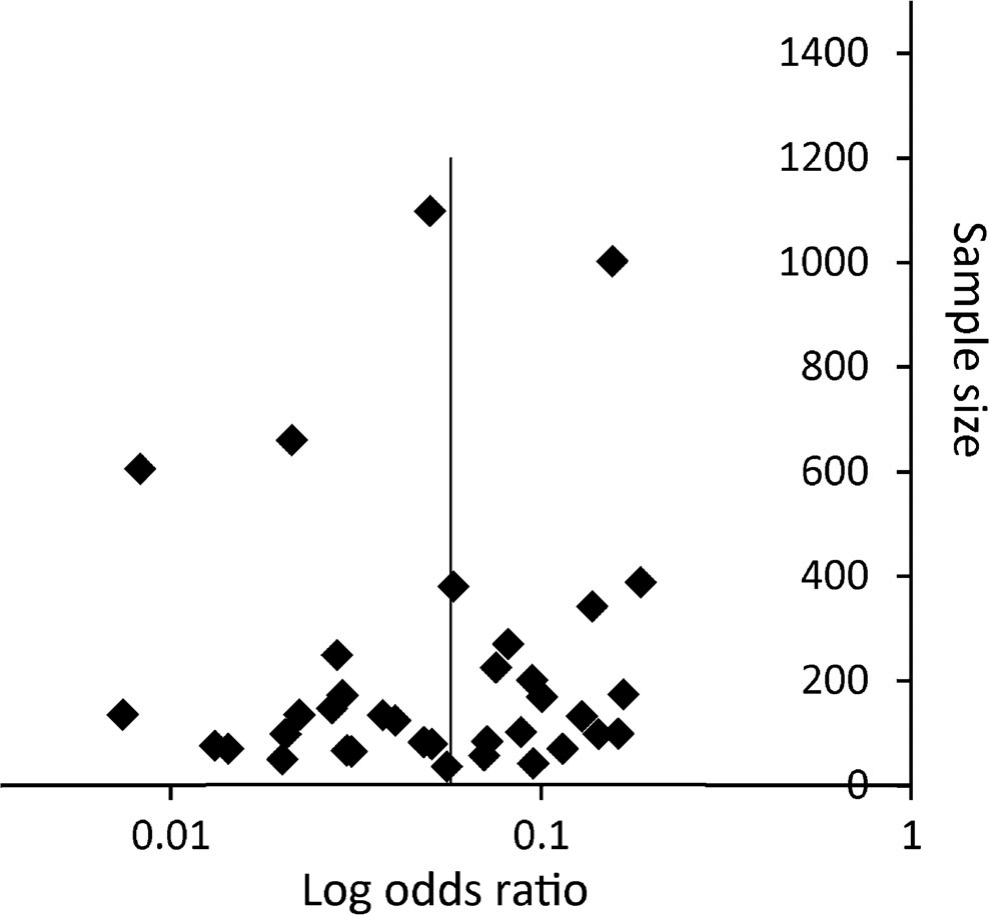
Funnel plot of major complications of CT-guided core biopsy

**Fig. 3:**
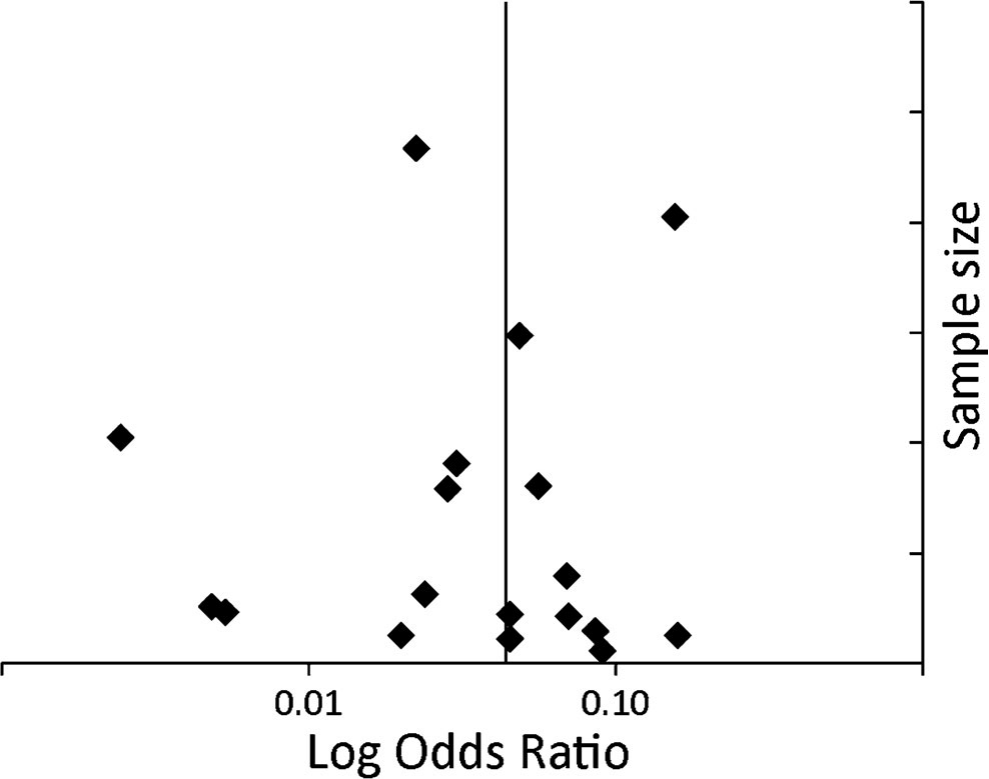
Funnel plot of major complications of CT-guided FNA

**Fig. 4:**
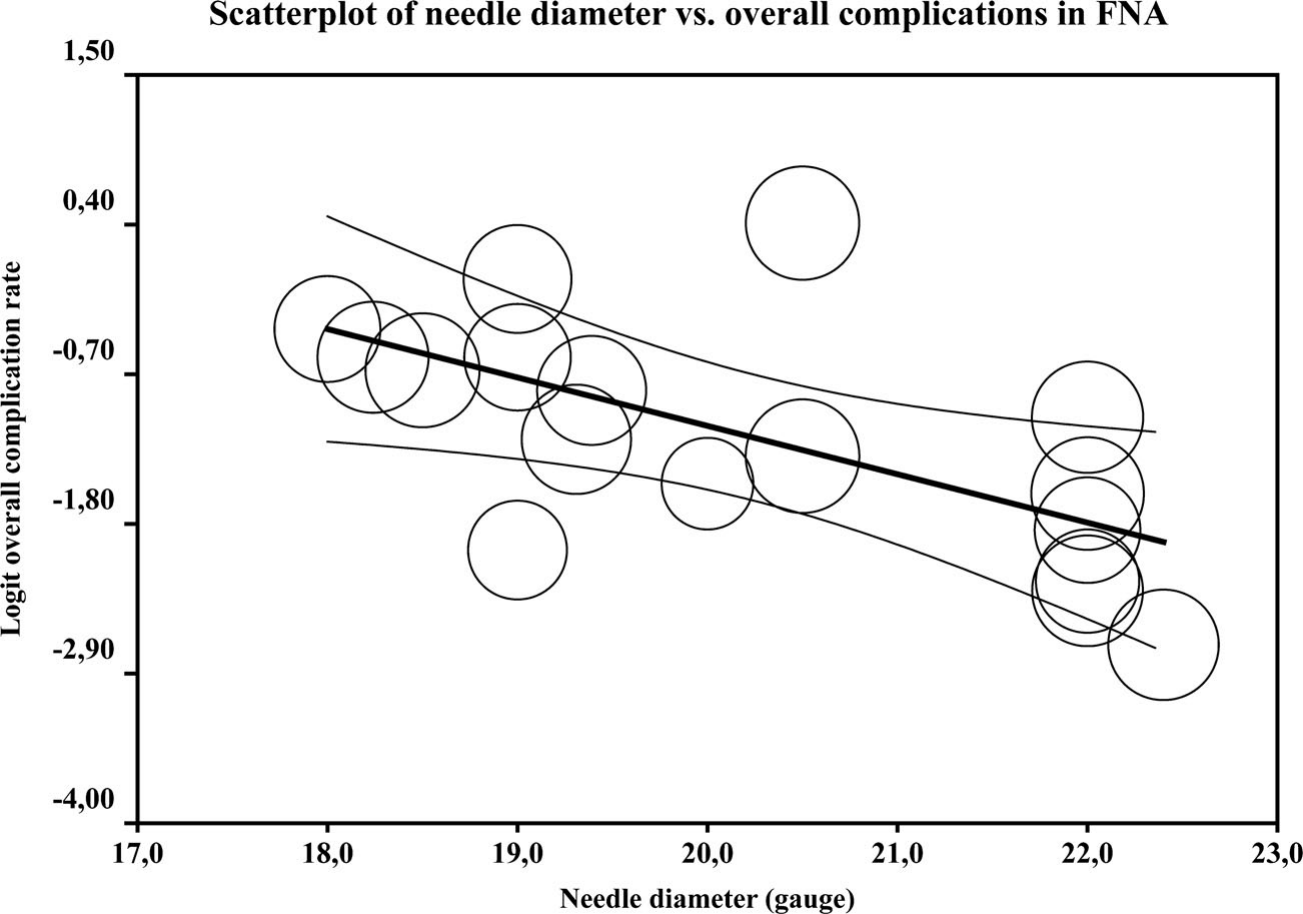
Scatter plot and regression line with 95 % confidence interval of mean needle diameter in relation to overall complication rate in FNA procedures. The size of the circles represents the relative weight of the study as assigned by the random effects model

The heterogeneity between studies was high; for core biopsy I^2^ = 93.74 % (Q-value 606.5, df (Q)= 38, *p* < 0.001) and for FNA I^2^ = 95.3 % (Q-value 362.3, df (Q)=17, *p* < 0.001). For core biopsy no sources of heterogeneity were identified. For FNA, needle diameter and nodule size were sources of heterogeneity, explaining 17 and 22 % of between-study variance, respectively. The funnel plots are shown in Figs. 2, 3, and 4 showing no indication of publication bias.

Table 5 shows the pooled complication rates of core biopsy and FNA, with respective odds ratios. Core biopsy had an overall complication rate of 38.8 % (95 % CI: 34.3–43.5 %), versus 24.0 % (95 % CI: 18.2–30.8 %) for FNA (*p* < 0.001). Respective rates for major complications were 5.7 % (95 % CI: 4.4–7.4 %) and 4.4 % (95 % CI: 2.7–7.0 %) (*p*=n.s.). In total, only five studies [30, 31, 34, 44, 50] reported cases of hemothorax; no pooled hemothorax rate could reliably be determined. Needle tract seeding, air embolism, and death were not reported in the included studies. The overall pneumothorax rate, the pulmonary haemorrhage rate, and the hemoptysis rate of FNA procedures were significantly lower than those of core biopsy procedures.

**Table 5:** Pooled complication rates of CT-guided transthoracic lung biopsy

	Complication rates (95 % confidence intervals)		Odds ratio^a^	*p*-value
	Core biopsy	FNA		
Pneumothorax	25.3 % (22.2–28.6 %)	18.8 % (14.6–23.9 %)	0.69 (0.50–0.96)	0.027
Pneumothorax intervention	5.6 % (4.3–7.3 %)	4.3 % (2.7–7.0 %)	0.76 (0.48–1.37)	0.430
Pulmonary haemorrhage	18.0 % (13.4–23.8 %)	6.4 % (2.5–15.2 %)	0.33 (0.15–0.72)	0.005
Hemoptysis	4.1 % (2.8–6.1 %)	1.7 % (0.9–3.1 %)	0.38 (0.17–0.85)	0.019
Overall complications	38.8 % (34.3–43.5 %)	24.0 % (18.2–30.8 %)	0.50 (0.35–0.73)	<0.001
Major complication	5.7 % (4.4–7.4 %)	4.4 % (2.7–7.0 %)	0.80 (0.48–1.36)	0.416

^a^Core biopsy is reference

### Risk factors for complications

Table 6 lists all risk factors for complications with a *p*-value < 0.20. For FNA procedures, larger needle diameter was a risk factor for overall complications, with an odds ratio of 0.70 (95 % CI: 0.55–0.89 %; *p* = 0.004) per gauge. When analyzing the needle diameter categorically, an FNA needle gauge of 22 or higher resulted in decreased odds of overall complications of 0.30 (95 % CI: 0.15–0.59 %; *p* < 0.001) compared to lower needle gauges. Figure 2 shows a scatter plot of the mean needle size in FNA studies against the complication rate. Increased mean lesion diameter decreased the risk of major complications (OR: 0.97 per mm; 95 % CI: 0.95–0.99 %; *p* = 0.017), and increased traversed lung parenchyma increased the risk of major complication (OR: 1.05 per mm; 95 % CI: 1.00–1.11 %; *p* = 0.035). The meta-analysis of core biopsies did not reveal significant risk factors for overall complications or for major complications.

**Table 6:** Risk factors for complications and major complications for CT-guided transthoracic core biopsy and FNA of lung lesions

	Studies (n)	Procedures (n)	Odds ratio (95 % CI)	*p*-value
Core biopsy: Overall complications				
Coaxial needle	32	8,133	1.37 (0.88–2.12)	0.164
Mean lesion size	32	8,133	0.98 (0.94–1.01)	0.185
Use of biopsy device	32	8,133	1.44 (0.91–2.27)	0.115
Core biopsy: Major complications				
CT-fluoroscopy	32	8,133	1.62 (0.81–3.23)	0.171
Mean patient age	32	8,133	1.09 (0.98–1.21)	0.124
FNA: Overall complications				
Mean needle gauge	17	4,084	0.70 (0.55–0.89)	0.004
Needle diameter >= 22 gauge	17	4,084	0.30 (0.15–0.59)	<0.001
Mean lesion size	12	2,357	0.97 (0.93–1.00)	0.073
Mean number of biopsy samples	6		1.36 (0.50–3.71)	0.549
FNA: Major complications				
Mean needle gauge	17	4,084	0.82 (0.64–0.96)	0.106
Needle diameter >= 22 gauge	17	4,084	0.58 (0.27–1.27)	0.172
Traversed lung (mm)	4	797	1.05 (1.00–1.11)	0.035
Coaxial needle	17	4,084	1.87 (0.80–4.33)	0.146
Lesion size (mm)	12	2,357	0.97 (0.95–0.99)	0.017
Number of operators	10	2,496	1.29 (0.97–1.71)	0.079

Only potential risk factors with a significant of *p* < 0.20 are listed in this table

## Discussion

This meta-analysis determined the complication rate of CT-guided core biopsy and FNA procedures and identified risk factors for complications. For CT-guided core biopsy the pooled rate of pneumothorax was 25.3 %, of pneumothorax requiring intervention 5.6 %, of pulmonary haemorrhage 18.0 %, and of hemoptysis 4.1 %. For FNA procedures these rates were lower, 18.8, 4.3, 6.4, and 1.7 %, respectively. This difference was significant for all minor complications. For FNA, larger needle diameter, smaller lesion size, and increased traversed lung parenchyma were risk factors for complications.

To our knowledge, no prior meta-analysis has studied the complication rate of CT-guided lung biopsy. Two large studies based on multi-centre procedures have been published. Wiener et al. [54] estimated the complications rate of CT-guided lung biopsy by analyzing two North American databases containing 15,865 procedures and found that pneumothorax occurred in 15.0 %, pneumothorax requiring chest tube in 6.6 %, and pulmonary hemorrhage in 1.0 %. No distinction was made between core biopsy and FNA. Tomiyama et al. [55] published a survey of severe complications based on 9,783 CT-guided lung biopsies in Japan and found pneumothorax in 35 %. These results are quite discrepant. Neither of these studies was included in this meta-analysis, because they did not meet the inclusion criteria. In *Quality Improvement Guidelines for Percutaneous Needle Biopsy*, the SIR and ACR published an estimated pneumothorax rate of 12–45 % and a chest tube placement rate of 2–15 % [56]. Again, this is a wide range, without differentiation between core biopsy and FNA. However, our estimates of complication rate are approximately in the centre of their estimated range.

Hemothorax rate could not be estimated reliably, because it was reported in only six studies. Other rare major complications such as needle tract seeding, air embolism and death were not reported by any of the included studies. Non-included studies reported a range of 0.02–0.4 % for air embolism [55, 57], 0.012–0.061 % for needle tract seeding [55, 58], and 0.16 % for death [59]. In our selected studies (12,753 procedures in total) these complications could have been expected to occur, but such results are probably not as likely to be published. Therefore, an underrepresentation of these very rare and major complications is likely to exist in this meta-analysis.

Comparing core biopsy with FNA is not straightforward. Core biopsy and FNA have their own advantages when used to diagnose lung lesions. Still, overall, complications occurred less often in FNA procedures (OR: 0.50; 95 % CI: 0.35–0.73 %). For major complications this correlation was not significant, although a similar trend was visible in favour of FNA. Included studies that compared FNA with core biopsy [7, 8, 60] did not find significant differences in complication rate, and in a systematic review comparing FNA with core biopsy in lung cancer diagnosis, Yao et al. [9] reported inconsistent results concerning complication rates.

In this study, a smaller lesion size and an increased distance traversed through lung parenchyma were found as risk factors for major complications in case of FNA. Patient and nodule characteristics most often mentioned as risk factors are older age, presence of emphysema, smaller lesion size, increased lesion depth, non-pleural contact, and smaller pleural-needle angle [38, 39, 61–63]. However, in most included studies these characteristics are only reported as a mean, and complications are not stratified based on these variables. Therefore, this meta-analysis is not ideal to identify patient- and nodule-specific risk factors.

In contrast, a meta-analysis can find risk factors in study-specific characteristics such as needle size, use of coaxial needle, number of biopsies, use of CT-fluoroscopy, on-site cytology, number of operators, operator experience, and institute frequency—factors that would be hard to identify in a single-cohort/institute, retrospective study. There was no difference in risk of complication between high and low volume centres. Also, no significant correlation between the sample size, number of biopsies, number of operators, operator experience, use of coaxial needle, CT-fluoroscopy, on-site cytology, or biopsy site down technique and (major) complication rate was found.

Only papers using CT guidance (conventional and/or CT-fluoroscopy) were included in this study. Conventional CT guidance offers the advantage of a simulated 3D view making it easy to look along the needle path. Also, there is no ionizing radiation exposure for the operator. CT-fluoroscopy offers the advantages of a near real-time imaging feedback as the needle is being inserted. It is, however, associated with an increased patient and operator dose [22, 64, 65]. These methods can be used interchangeably, e.g. starting with conventional CT guidance and switching to CT-fluoroscopy when the lung lesion proves difficult to reach because of patient respiration. For core biopsy we found a trend that suggested CT-fluoroscopy might result in a higher major complication rate (OR: 1.62; 95 % CI: 0.81–3.23 %; *p* = 0.171). CT-fluoroscopy is generally reported to have a lower complication rate due to shorter procedure time and fewer needle passes [22, 33, 64]. However, if CT-fluoroscopy is indeed used more frequently in cases of hard-to-reach lesions, it could potentially bias the results, as it generally takes longer to sample these. Lastly, it should be noted that none of the included studies used CT-fluoroscopy for FNA.

Intuitively, operator experience is thought to influence complication rate. In a large single-cohort study, Yeow et al. [38] reported operator experience as the third major risk factor for pneumothorax. In our meta-analysis only a few papers reported the operator experience (core biopsy: *n* = 10; FNA: *n* = 3). Because of the low number of studies reporting operator experience, and because only overall mean operator experience was reported (so not per operator), it was not possible to further study this potential risk factor in our meta-analysis.

The use of coaxial needles has the advantage of decreasing the number of pleural passes. However, it results in a prolonged connection with the pleura which might lead to increased parenchymal damage due to respiratory motion. Also, it increases the outer needle diameter. None of the included studies specifically investigated the effect of coaxial needles on complication rate. Two other studies did [66, 67], but found no significant correlation. In meta-regression there were trends for coaxial needles towards an increase in overall complications for core biopsy (OR: 1.37; 95 % CI: 0.88–2.12 %; *p* = 0.164), and for FNA (OR: 1.87; 95 % CI: 0.80–4.33 %; *p* = 0.146), but none of these correlations were significant.

The biopsy site down technique has been cause for some debate; although some papers report no difference in complication rate when repositioning the patient after the procedure [64], others have demonstrated a considerable reduction in pneumothorax and/or chest tube placement rate in case of patient repositioning [65]. O’Neill et al. [66] suggested that the critical factor for success is to immediately roll the patient over after biopsy, calling it *rapid needle-out patient-rollover*. Kim et al. [67] recently reported a significant reduction of chest tube placement in cone beam CNB in a retrospective study among 1,191 patients, using similar technique. None of the papers included in this meta-analysis, using the biopsy site down technique, mentioned to roll the patients over immediately, which is why this correlation could not be evaluated. However, Kinoshita et al. [26] have been positioning patients (*n* = 147) with the biopsy site downwards during the procedure, using a special table, after which they stayed in biopsy site down position for approximately 15 min. They report a considerable drop in pneumothorax rate compared to the standard procedure, which also suggests that patient (re) positioning in the initial minutes after, or even during, biopsy is critical.

For overall complications in FNA, the use of larger needles was a risk factor. Per increased needle gauge, the risk decreased by 30 % (OR: 0.70; 95 % CI: 0.55–0.89 %). According to the guidelines of the SIR [56] only procedures performed with 22 or higher gauge needles should be considered *fine* needle aspiration. When categorized according to this definition, the use of fine needles compared to larger needles decreased the risk of complications by 70 % (OR: 0.30; 95 % CI: 0.15–0.59 %). For major complications, however, needle size was not a significant risk factor. For core biopsy procedures no significant risk factors were found.

Studies were only included in this meta-analysis if they reported adequate monitoring of complications. Although that resulted in the exclusion of 75 studies, it made sure that no studies were included that underreported their complication rate. Because chest radiography has demonstrated to miss a significant number of pneumothorax cases after CT-guided lung biopsy compared to CT [68], an initial control CT scan was a requirement for inclusion. Also, at least additional chest radiography 2 to 4 h after the procedure was required, because studies have shown that initially covert pneumothorax detected by delayed chest radiograph sometimes does require chest tube insertion [49, 69]. Another inclusion criterion was the reporting of complications of at least 50 procedures, resulting in the exclusion of 32 studies. Excluding smaller studies may bias results, as less experienced departments can be expected to have a higher complication rate. The funnel plots of included studies show no clear asymmetry, and regression analysis showed no correlation between sample size and complication rate, so we do not expect the exclusion of small studies to have biased the results. However, since small studies were not included in the analyses, this cannot be excluded with certainty.

We investigated potential sources of heterogeneity, but much of the variance between studies could not be explained. Therefore, a random-effects model was used to pool complication rates. This makes the estimates more reliable as it favours larger studies relatively less, compared to a fixed effects model. Also, outliers do not get weighted as heavily as they otherwise would. Overall, we expect that the provided pooled complication rates are accurate estimates of actual complication rates.

This study has some limitations. It was not designed to compare specifically the complication rates of core biopsy with FNA. Most included studies only report complications of one method, so usually no controls in the same population are available. Also, although the quality of the studies included in both groups is generally high according to the NOS score, there are potential sources of bias within the studies. In the past, FNA would be preferred over core biopsy for small nodules, because core samples of high quality were considered hard to obtain. It has previously been shown that smaller lesions are more likely to result in complications, which might cause the pooled complication rates of core biopsy and FNA to be biased [24, 40]. However, in our meta-analysis the mean lesion diameter for core biopsy was significantly smaller than for FNA procedures (28 vs. 42 mm). Although lesion size was not a significant risk factor for complications of core biopsy, this difference could be a potential confounder. Another potential source of within study bias could be in the selection of sampling techniques in case additional histological subtyping is required for targeted therapy. In those cases core biopsy is often preferred, and these patients can be expected to have a higher comorbidity resulting in a higher complication rate.

Papers were only included if complications of FNA and core biopsy were presented separately. Sometimes both techniques are used in the same setting in an effort to increase the diagnostic performance, which can inadvertently lead to a higher rate of complications [70–72]. This study cannot draw conclusions as to whether or not combined usage of sampling techniques indeed increases the complication rate.

Lastly, this study could not determine the rate of complications that occur infrequently or require a long term follow-up of the patient, such as death, air embolism and needle tract seeding.

In the specific context of diagnostic work-up of lung nodules detected in CT-screening FNA should be favoured over core biopsy. This is especially the case for 22-gauge needles, with which the risk of complications decreases greatly. Also, studies have shown that diagnostic yield does not decrease when using smaller FNA needles [73], and advances in FNA cytology have enabled subtyping of lung cancer in cytological material [74].

It should be considered that a smaller lesion size is a risk factor for major complications, and that nodules detected by lung cancer CT-screening, needing work-up, are generally smaller in size [75]. However, factors such as younger age and less comorbidity that can be expected in screening patients will have a beneficial effect on the expected complication rate. Overall, the pooled complication rate determined in this meta-analysis cannot be assumed to be similar in screen-detected nodules.

In order to compare the complications of core biopsy and FNA properly, only randomized controlled trials or even only prospective studies comparing both techniques should be included in the meta-analysis. However, randomized controlled trials comparing these techniques have not been published, and only two prospective studies compared complication rates. Therefore, well-designed randomized controlled trials would be recommended to definitively compare the safety of CT-guided lung core biopsy and FNA.

## Conclusion

For CT-guided lung biopsy the overall complication rate is acceptable and the major complication rate is low. Minor complications occur more often with core biopsy compared to FNA. For major complications this difference is not significant. In cases of FNA, larger needle size is a risk factor for overall complications, and risk factors for major complications are smaller lesion size and increased traversed lung parenchyma. CT-guided lung biopsy, and particularly FNA with small needles, can be an important diagnostic tool, with a low major complication rate.

## Electronic supplementary material

Below is the link to the electronic supplementary material.

ESM 1 (DOCX 119 kb)

## Abbreviations

95 % CI: 95 % Confidence interval
CT: Computed tomography
FNA: Fine needle aspiration
NOS: Newcastle Ottawa scale
OR: Odds ratio
SIR: Society of Interventional Radiology
