# Supplementary material for: Complication rates of CT-guided transthoracic lung biopsy: meta-analysis
Source: Eur Radiol. 2016 Apr 23;27(1):138–48. doi: 10.1007/s00330-016-4357-8 (PMC5127875; doi:10.1007/s00330-016-4357-8)
Supplement: Supplementary file 1 — (DOCX 119 kb) [file 330_2016_4357_MOESM1_ESM.docx]

## Supplemental material

E-Table 1 Literature search strategy. Last search on August 21, 2015

| **Database** | **Search query** | **Results** |
| --- | --- | --- |
| PubMed | #1: (Biopsy[mesh] OR “biopsy, fine-needle”[mesh] OR “Needle Biopsy”[mesh] OR biopsy[tiab] OR FNA[tiab] OR “fine needle aspiration”[tiab])  #2: (transcutane*[tiab] OR percutane*[tiab] OR transthorac*[tiab] OR “CT FNA”[tiab] OR “CT-guided”[tiab] OR “CT guided”[tiab] OR “CT fluoroscopy-guided”[tiab] OR “computerized tomography-guided”[tiab])  #3: (“Lung Neoplasms”[mesh] OR nodule* OR pulmonary OR lung)  #4: (“Multidetector Computed Tomography”[mesh] OR “Computed Tomography, X-Ray”[mesh] OR CT[tiab] OR “computed tomography”[tiab] OR “computed tomogram”[tiab])  Grammar in advanced search: #1 AND #2 AND #3 AND #4 | 1.647 |
| Embase | #1: (biopsy OR 'needle biopsy' OR 'fine needle aspiration biopsy' OR fna:ab,ti)  #2: (transcutane* OR percutane* OR transthoracic* OR 'ct fna' OR 'ct-guided' OR 'ct guided' OR 'ct fluoroscopy-guided':ti,ab)  #3: ('lung cancer' OR 'lung tumor' OR nodule* OR lung:ab,ti OR pulmonary:ab,ti)  #4: ('computer assisted tomography' OR 'computed tomography' OR ct OR 'computed tomogram' OR 'computerized tomography')  Grammar in advanced search: #1 AND #2 AND #3 AND #4 | 3.160 |
| Web of Science | (TS=(Biopsy OR fine-needle biopsy OR FNA OR fine needle aspiration) OR TI = (biopsy OR fna OR "fine needle aspiration")) AND  TI=(transcutane* OR percutane* OR transthorac* OR "CT FNA" OR "CT-guided" OR "CT fluoroscopy-guided" OR "computerized tomography-guided") AND  TS=(lung cancer OR lung neoplasms) OR TI=(nodule* OR pulmonary OR lung) AND  TS=(multidetector computed tomography OR computed tomography OR CT OR computed tomogram) OR TI=(computed tomography OR CT OR computed tomogram OR computerized tomography) | 483 |
| Cochrane Library | #1: biopsy or "fine needle biopsy" or FNA or "fine needle aspiration"  #2: transcutane* or percutane* or transthorac* or thorac* or “ct fna” or “ct-guided” or “ct guided” or "ct fluoroscopy-guided"  #3: "lung neoplams" or nodule* or pulmonary or lung or “lung cancer”  #4: "computed tomography" OR ct OR "computed tomogram"  Grammar: #1 AND #2 AND #3 AND #4 | 52 |

E-tables 2-9 are in EMF format to prevent resolution loss when rescaled. Other formats available upon request.

E-table 2: Forest plot of pneumothorax rate in core biopsy studies

E-table 3: Forest plot of major pneumothorax rate in core biopsy studies

E-table 4: Forest plot of hemoptysis rate in core biopsy studies

E-table 5: Forest plot of pulmonary hemorrhage rate in core biopsy studies

E-table 6: Forest plot of pneumothorax rate in FNA studies

E-table 7: Forest plot of major pneumothorax rate in FNA studies

E-table 8: Forest plot of hemoptysis rate in FNA studies

E-table 9: Forest plot of pulmonary hemorrhage rate in FNA studies
